# Hospital-Associated Transmission of Candida auris from Adult to Pediatric Patient

**DOI:** 10.1017/ash.2024.230

**Published:** 2024-09-16

**Authors:** Erin Barker, Vivian Donnelly, Susan Fallon, Sara Pau, Melanie Curless, Erica Prochaska, Shannon Murphy, Patricia Simner, Sean Zhang, Aaron Milstone, Anna Sick-Samuels

**Affiliations:** The Johns Hopkins Hospital; Johns Hopkins University; Johns Hopkins School of Medicine

## Abstract

**Background:** Candida auris, an emerging multidrug-resistant fungus, is often difficult to control in hospital outbreaks. We report the hospital investigation and findings of a transmission of C. auris from patients hospitalized in an adult unit to a pediatric unit, the first in Maryland. **Methods:** Between June and September 2023, C. auris was recovered from two patients admitted to an adult Neuroscience Intensive Care Unit (ICU) and a patient admitted to a pediatric ICU. Infection control initiated an investigation involving staff interviews, observations and chart reviews. Cases were defined as any patient with clinical or surveillance cultures growing C. auris. Point prevalence surveillance was conducted by collecting nares and composite axilla/groin swabs from patients on the affected units. Environmental cultures collected using moistened E-Swabs (Copan, Murrieta, CA) from shared supplies were plated on CHROMagar Candida (BD, Sparks, MD). C. auris isolates from patients hospitalized at the facility between February 2022 and October 2023 were analyzed by WGS for relatedness. WGS was performed using Illumina NextSeq 300 bp paired-end sequencing (Illumina, San Diego, CA). Single nucleotide polymorphism (SNP) analysis was performed by comparing raw reads to the reference C. auris B8441 genome for subsequent clustering analysis (Ares Genetics, Vienna, Austria). **Results:** WGS demonstrated isolates from two adults and one pediatric patient were less than three SNPs different, suggesting a shared isolate. One additional pediatric case was identified from surveillance cultures collected from 27 patients. Investigation into possible transmission routes revealed healthcare personnel serving both units, specifically clinical teams and continuous electroencephalography (cEEG) technologists. Additionally, cEEG equipment was used on both adult and pediatric patients and twelve equipment surface swabs and three samples each of measuring tape and gel were collected. C. auris was not isolated, however sensitivity of environmental sampling is unclear and suspicion for possible fomite/environmental transmission persisted. Other possible transmission routes included gaps in hand hygiene, isolation, disinfection of shared equipment, and reuse of single-use items. Interventions included improving and monitoring infection prevention practices, educating multi-disciplinary personnel and heightened environmental cleaning. **Conclusion:** This case highlights the feasibility of transmission of C. auris between patients admitted to a geographically distant unit. Our investigation revealed multiple possible routes of transmission including direct contact (from healthcare personnel or equipment) or indirect environmental sources. Prevention of hospital-associated C. auris transmission likely necessitates meticulous adherence to hand hygiene, contact precautions, and careful cleaning and disinfection of patient environments and equipment used by all disciplines.

**Disclosure:** Patricia Simner: Research Contracts: BD Diagnostics, OpGen Inc., Qiagen Sciences Inc, T2 Diagnostics, Accelerate Diagnostics; Research Collaborators:Ares Genetics, CosmosID, IDbyDNA, Illumina; Consulting: OpGen Inc., BD Diagnostics, Shionogi Inc., GeneCapture, Qiagen Sciences Inc, Entasis, Day Zero Diagnostics, Next Gen Diagnostics

